# Factors Associated with *Legionella* Detection in the Water Systems of National Lodging Organization Facilities with Water Management Programs in the United States

**DOI:** 10.3390/ijerph21070939

**Published:** 2024-07-18

**Authors:** Rebecca Kahn, Gordana Derado, Elizabeth J. Hannapel, Patrick Vander Kelen, Jasen M. Kunz, Chris Edens

**Affiliations:** 1 Division of Bacterial Diseases, National Center for Immunization and Respiratory Diseases, Centers for Disease Control and Prevention, Atlanta, GA 30329, USA; uwx8@cdc.gov (G.D.); qlr0@cdc.gov (E.J.H.); iek4@cdc.gov (C.E.); 2 Epidemic Intelligence Service, Centers for Disease Control and Prevention, Atlanta, GA 30329, USA; 3 Division of Environmental Health Science and Practice, National Center for Environmental Health, Centers for Disease Control and Prevention, Atlanta, GA 30329, USA; lup8@cdc.gov; 4 Division of Foodborne, Waterborne, and Environmental Diseases, Centers for Disease Control and Prevention, Atlanta, GA 30329, USA; izk0@cdc.gov

**Keywords:** *Legionella*, Legionnaires’ disease, water management programs, national lodging organization

## Abstract

A better understanding of risk factors and the predictive capability of water management program (WMP) data in detecting *Legionella* are needed to inform the efforts aimed at reducing *Legionella* growth and preventing outbreaks of Legionnaires’ disease. Using WMPs and *Legionella* testing data from a national lodging organization in the United States, we aimed to (1) identify factors associated with *Legionella* detection and (2) assess the ability of WMP disinfectant and temperature metrics to predict *Legionella* detection. We conducted a logistic regression analysis to identify WMP metrics associated with *Legionella* serogroup 1 (SG1) detection. We also estimated the predictive values for each of the WMP metrics and SG1 detection. Of 5435 testing observations from 2018 to 2020, 411 (7.6%) had SG1 detection, and 1606 (29.5%) had either SG1 or non-SG1 detection. We found failures in commonly collected WMP metrics, particularly at the primary test point for total disinfectant levels in hot water, to be associated with SG1 detection. These findings highlight that establishing and regularly monitoring water quality parameters for WMPs may be important for preventing *Legionella* growth and subsequent disease. However, while unsuitable water quality parameter results are associated with *Legionella* detection, this study found that they had poor predictive value, due in part to the low prevalence of SG1 detection in this dataset. These findings suggest that *Legionella* testing provides critical information to validate if a WMP is working, which cannot be obtained through water quality parameter measurements alone.

## 1. Introduction

The bacterium *Legionella* can cause diseases, ranging from severe pneumonia (i.e., Legionnaires’ disease (LD)) and the rarer but severe extrapulmonary legionellosis, to the milder respiratory illness of Pontiac fever. More than 95% of reported case-patients with LD in the United States are hospitalized, and approximately 10% result in death [1]. Outbreaks of LD are often linked to poorly maintained water systems in settings such as hotels and healthcare facilities. The Centers for Disease Control and Prevention (CDC) and ASHRAE (formerly known as the American Society of Heating, Refrigerating and Air-Conditioning Engineers) recommend facilities with certain characteristics or devices develop and implement water management programs (WMPs) to help reduce the risk of *Legionella* growth/spread and to reduce outbreaks [2,3]. WMPs include the establishment of controls, such as disinfectant residuals and temperatures, as well as measurements, to ensure that values are within control limits. The US Department of Veterans Affairs and the Centers for Medicare and Medicaid Services have WMP requirements.

Testing building water systems and devices for *Legionella* has several purposes, including establishing baseline measurements, assessing the impact of remedial measures, and investigating sources of exposure during public health investigations of the disease [4]. In addition, testing is a way to confirm that a WMP is effectively working (validation) [2,3,5,6,7]. The CDC has outlined multifactorial indicators for routine *Legionella* test result interpretation for WMP performance, including the concentration of *Legionella* and associated trends over time, the extent of *Legionella* growth (i.e., one vs. multiple sources), the location of detection within the water system, and the *Legionella* species’ association with LD [4,6].

A better understanding of risk factors and the predictive capability of WMP data in detecting *Legionella* are needed to inform future efforts to reduce *Legionella* growth and prevent outbreaks. In this analysis, by using WMP and *Legionella* testing data from a national lodging organization (NLO) with over 700 lodging facilities located in the United States, we aimed to (1) identify factors associated with *Legionella* detection and (2) assess the ability of WMP metrics to predict *Legionella* detection.

## 2. Materials and Methods

### 2.1. Data

The NLO shared 2018–2020 WMP and *Legionella* testing data, the details of which have been described elsewhere [8]. The NLO requires each facility to have a WMP and to conduct at least annual environmental testing for *Legionella* by traditional spread-plate culture from the building’s hot or cold water premise plumbing system. Positive culture results were categorized as *Legionella pneumophila* serogroup 1 (SG1) or non-SG1. The WMP data include free and total disinfectant levels, grouped into categorical levels, from primary test points in hot water (i.e., tap most distal from the water heater within the hot water distribution system), cold water (i.e., tap closest to the building’s incoming water main), supply temperature data (i.e., water leaving the water heater), and return temperature data (i.e., hot water recirculation systems prior to reheating).

### 2.2. Outcome

Because SG1 is more strongly associated with disease than other serogroups and species [9], we define our primary outcome variable as binary SG1 detection (yes/no). When facilities conducted multiple tests per day, the results were aggregated by day for each facility (i.e., any positive test vs. none on that day), keeping tests that came from hot vs. cold water systems separate. In a sensitivity analysis, we also examined any *Legionella* detection (SG1 and/or non-SG1, i.e., *Legionella pneumophila* serogroups other than SG1 or other species of *Legionella*) as the outcome.

### 2.3. WMP Failure Metrics

For each day that *Legionella* testing occurred in a facility, we created six binary WMP failure metrics to identify if a failure had been detected at any point in the five weeks prior to the testing date. The failure metrics included any failure in the past five weeks in the following: (1) the return water temperature in guest rooms, (2) the supply water temperature in guest rooms, (3) the primary test point for total disinfectant levels in cold water, (4) free disinfectant levels in cold water at the primary test point, (5) the primary test point for total disinfectant levels in hot water, and (6) free disinfectant levels in hot water at the primary test point (Table 1). We used a five-week timeframe to balance temporality (i.e., addressing failures that occurred too far in the past) and sample size (because disinfectant and temperature testing were not conducted every week at every facility).

We define failure based on control limits established in the NLO policy, which include a control limit for a return temperature of ≤118 °F and a supply temperature of <124 °F. For free disinfectant levels, the control limits in hot and cold water are less than 0.4 mg/L and 0.5 mg/L, respectively, and for total disinfectant, the control limits in hot and cold water are less than 1.0 mg/L each. We exclude any reported temperature values above 165 °F, as those are likely incorrect (<1% of total values; see [8] for more details on WMP data).

### 2.4. Statistical Analysis

We conducted a mixed-effects logistic regression analysis with random intercepts for the facility to identify WMP metrics associated with SG1 detection. We tested models with different combinations of the six WMP failure variables, using the Akaike information criterion (AIC), Bayesian information criterion (BIC), and the likelihood ratio test (for nested models) to determine the best fitting model. In all models considered, we also controlled for the hot vs. cold water system, the season—to account for the seasonality of LD (high: February–July vs. low: August–January), and year (2019 vs. 2018 and 2020 vs. 2018). Variables were considered statistically significant at alpha = 0.05 if their 95% confidence intervals did not include 1. We also assessed the interactions between temperature and disinfectant failures.

### 2.5. Predictive Measures

We calculated the sensitivity, specificity, positive predictive value (PPV), and negative predictive value (NPV) for each WMP failure metric and SG1 detection (as well as any *Legionella* detection, SG1 or non-SG1) as part of our sensitivity analyses. Sensitivity was calculated as the percentage of times the WMP metric indicated a failure when there was SG1 detection; specificity was calculated as the percentage of times the WMP metric did not indicate a failure when there was no SG1 detection; PPV was calculated as the proportion of times there was SG1 detection when the WMP metric indicated a failure, and NPV was calculated as the proportion of times there was no SG1 detection when the WMP metric did not indicate a failure.

## 3. Results

After aggregating daily data by the facility and water system, there were 5435 testing observations in the dataset from 2018 to 2020 (January 2018–November 2020; building operations consisted of low or no hotel occupancy during the COVID-19 pandemic). Of these observations, 411 (7.6%) had SG1 detection from 85 of the 725 facilities (12%), and 1606 (29.5%) had either SG1 or non-SG1 detection from 230 facilities (32%). Each WMP metric was available in the five weeks prior to each *Legionella* test for approximately 75% of *Legionella* tests (Appendix A).

In the best fitting model based on the AIC, which included three of the WMP failure metrics, the following variables were significantly associated with SG1 detection: failure regarding total disinfectant levels in hot water, samples collected from the hot water system (vs. cold), and samples collected in 2020 (vs. 2018) (Table 2; see Appendix B and Appendix C for the other models considered). Failure at the primary test point for total disinfectant levels in hot water had the strongest association with SG1 detection: any failure in the past five weeks had 4.1 times the odds of SG1 detection (95% CI: 2.0, 8.5), compared to no failure in the past five weeks. Any failure in the past five weeks in the return water temperature in guest rooms had a positive but non-significant association with SG1 detection (OR = 1.6, CI = (0.8, 3.2)). The cooccurrence of failures at both primary test points for total disinfectant levels in hot and cold water was more common than the cooccurrence of failures in guest room return temperature with failures at either primary test point for total disinfectant levels (Appendix D). Interactions between temperature and disinfectant failures were not significant and were, therefore, not included in the final model.

Despite the associations identified between WMP failures and SG1 detection, failures in WMP metrics were not strong predictors of SG1 detection (Table 3, Appendix A). Consistent with the strong association observed, the failure sensitivity in the past five weeks at the primary test point for total disinfectant levels in hot water for SG1 detection was 87.7%. However, the PPV was only 5.6%, meaning that most of the time when this failure occurred, there was no SG1 detection.

We found qualitatively similar results when using any *Legionella* detection as the outcome, with slightly higher PPV; this finding was expected, given the higher percentage of positivity for SG1 or non-SG1 compared to SG1 only (Appendix E).

## 4. Discussion

With facility-level WMP metrics and *Legionella* test results, this dataset from an NLO provides detailed insights into factors associated with *Legionella* detection, which can help to inform the development of WMPs and *Legionella* testing practices. We found failures in commonly collected WMP metrics, particularly at the primary test point for total disinfectant levels in hot water, to be associated with SG1 detection. A similar analysis conducted in Veterans Health Administration healthcare buildings also found hot water samples and lower residual biocide concentrations to be associated with *Legionella* detection [10]. These findings highlight that WMPs may be important for reducing the risk of *Legionella* growth and subsequent disease; moreover, it may be important to regularly monitor water quality parameters, such as disinfectant levels and temperature, to ensure they are within expected ranges.

However, while unsuitable water quality parameter results are associated with *Legionella* detection, this study found that they had poor predictive value for *Legionella* detection. This finding is not surprising due in part to the low prevalence of SG1 detection in this dataset, as positive predictive value is influenced by prevalence; this means that when prevalence is low, failures in WMP metrics have less ability to predict *Legionella* detection than when prevalence is higher. Similarly, as expected with low prevalence, the NPV is much higher than the PPV, meaning that suitable water quality parameters suggest a decreased likelihood of *Legionella* detection, but they do not guarantee that *Legionella* growth is well-controlled.

The odds of *Legionella* detection were significantly higher in 2020 than in 2018, potentially reflecting the impacts of reductions in building occupancy and water usage during the COVID-19 pandemic. The impact of COVID-19 on WMP performance was described in detail in our recent study [8].

Several limitations of the NLO data have been described in detail elsewhere [8]. For these analyses, limitations to the data include a lack of information on the geography of where facilities are located (due to identifiability concerns), facility age, or occupancy fluctuations due to seasonal use, building closures, or renovations that occurred during the pre-pandemic months, all of which could potentially impact the odds of *Legionella* detection. Additionally, the disinfectant data did not specify the type of disinfectant used and were provided categorically, with zero disinfectants included in the category with the lowest levels. The number of data points by facility also varied greatly, although we attempted to account for this in our analytical approach. As described earlier, defining failures based on reported WMP data in the five weeks prior to a *Legionella* test was attempted to balance temporality and sample size. However, little prior research exists to determine the optimal window for the impact of WMP measures; in the present study, WMP measurements were not available for about 25% of the samples (Appendix A). Given the varying frequencies of testing for disinfectant and temperature levels, failures could have gone undetected. However, analyses of WMP failure metrics using data from two, three, and four weeks showed similar trends to the analysis using five weeks of data, strengthening our conclusions. Finally, due to the limitations in the *Legionella* data [8], such as variations in sampling and testing techniques that can impact the reliability of *Legionella* concentration data, we were not able to use the CDC’s multifactorial approach (as described earlier) [4] to interpret routine testing results. Therefore, we could not assess whether each sampling event was consistent with a system in which *Legionella* growth was well-controlled, poorly controlled, or uncontrolled.

## 5. Conclusions

This analysis builds on previous analyses [8], which found increased odds of SG1 detection during the COVID-19 pandemic compared to 2018–2019, with increased positivity driven by facilities that also had *Legionella*-positive sample results before the pandemic. Those findings suggested the NLO’s flushing protocols may have prevented some *Legionella* growth, but that additional control measures may be needed for some facilities. In both studies, we find that—while they remain an important part of any WMP—water quality parameter results that meet control limits do not guarantee that *Legionella* growth is well-controlled. These findings suggest that *Legionella* testing provides critical information to validate if a WMP is working, which cannot be obtained through water quality parameter measurements alone.

## Figures and Tables

**Table 1:** Failure variables and definitions.

Variable No.	Variable Name	Description	Failure (as Defined by WMP)
1	Return water temperature in guest rooms	Hot water recirculation system prior to reheating	≤118 °F
2	Supply water temperature in guest rooms	Water leaving the water heater	<124 °F
3	Primary test point for total disinfectant in cold water	Tap closest to the building’s incoming water main	<1.0 mg/L
4	Free disinfectant in cold water at the primary test point		<0.5 mg/L
5	Primary test point for total disinfectant in hot water	Tap most distal from the water heater within the hot water distribution system	<1.0 mg/L
6	Free disinfectant in hot water at the primary test point		<0.4 mg/L

**Table 2:** Odds ratios comparing SG1 detection to failure to detect *.

Variable	OR (95% CI)
Any failure in the past five weeks in the return hot water temperature in guest rooms (variable no. 1)	1.6 (0.8, 3.2)
Any failure in the past five weeks at the primary test point for total disinfectant levels in cold water (variable no. 3)	0.9 (0.5, 1.6)
Any failure in the past five weeks at the primary test point for total disinfectant levels in hot water (variable no. 5)	4.1 (2.0, 8.5) *
Sample collected from the hot water system	2.3 (1.5, 3.7) *
Sample collected in 2020	2.4 (1.4, 4.3) *
Sample collected in 2019	1.2 (0.7, 2.3)
Sample collected in 2018	ref
Sample collected in the high season	0.8 (0.3, 1.9)

* significant, at alpha = 0.05.

**Table 3:** Performance metrics.

No.	Variable (Any Failure in the Past Five Weeks)	PPV	NPV	Sensitivity	Specificity
1	Return temperature in guest room failure	5.9%	96.1%	27.6%	80.3%
2	Guest room supply temperature failure	5.8%	95.7%	7.7%	94.3%
3	Primary test point for total disinfectant failure in cold water	6.4%	96.8%	48.9%	68.4%
4	Free disinfectant failure in cold water at the primary test point	5.3%	96.6%	54.0%	57.1%
5	Primary test point for total disinfectant failure in hot water	5.6%	98.5%	87.7%	35.9%
6	Free disinfectant failure in hot water at the primary test point	5.3%	97.0%	63.7%	50.2%

## Data Availability

Restrictions apply to the availability of these data. Data were obtained from a private entity and are available from the authors with the permission of the NLO and in accordance with the data use agreement(s).

## Appendix A

	**Any Failure in the Past Five Weeks in Return Temperature in Guest Room (Variable No. 1) ***		
**SG1 Detection**	**No Fail**	**Fail**	**Total**
No	3132	768	3900
Yes	126	48	174
Total	3258	816	4074
* Available for 75% (4074/5435) of testing observations			
	Any failure in the past five weeks in guest room supply temperature (variable no. 2) *		
SG1 detection	No fail	Fail	Total
No	3765	228	3993
Yes	169	14	183
Total	3934	242	4176
* Available for 77% (4176/5435) of testing observations			
	Any failure in the past five weeks at the primary test point for total disinfectant in cold water (variable no. 3) *		
SG1 detection	No fail	Fail	Total
No	2716	1253	3969
Yes	90	86	176
Total	2806	1339	4145
* Available for 76% (4145/5435) of testing observations			
	Any failure in the past five weeks at the primary test point for free disinfectant in cold water (variable no. 4) *		
SG1 detection	No fail	Fail	Total
No	2267	1702	3969
Yes	81	95	176
Total	2348	1797	4145
* Available for 76% (4145/5435) of testing observations			
	Any failure in the past five weeks at the primary test point for total disinfectant in hot water (variable no. 5) *		
SG1 detection	No fail	Fail	Total
No	1416	2528	3944
Yes	21	150	171
Total	1437	2678	4115
* Available for 76% (4115/5435) of testing observations			
	Any failure in the past five weeks at the primary test point for free disinfectant in hot water (variable no. 6) *		
SG1 detection	No fail	Fail	Total
No	1979	1967	3946
Yes	62	109	171
Total	2041	2076	4117
* Available for 76% (4117/5435) of testing observations			

## Appendix B. Random Intercept Model Choice

We chose the mixed effect modeling approach, with a random intercept for the facility to account for the correlation between repeat measures at the same facility. We also considered using a generalized estimating equation (GEE) approach. However, to properly model the correlation in a GEE model, the data need to be aggregated up to months or quarters due to the wide variations in the number of repeated measurements across facilities. This aggregation makes interpretation more challenging and temporality less clear. We also considered using Poisson instead of logistic regression models, with a percent positive rate, to account for repeated testing on the same day (or month, if aggregated). This approach was challenging due to the high proportion of zeros (i.e., negative tests), and there were some model fitting and convergence issues with zero-inflated models.

## Appendix C. Random Intercept Modeling

We tested models with different combinations of the six WMP failure variables. Model 1 included all WMP failure variables, and the subsequent models removed variables based on the bivariate AICs or subject matter knowledge (e.g., Model 4 was selected based on AIC but Model 5 included both hot and cold water disinfectant failures). The final model was chosen based on the model metrics. In all models considered, we also controlled for the hot vs. cold water system, season (high vs. low), and year (2019 vs. 2018 and 2020 vs. 2018).

**Model**	**Variables**	**OR (95% CI)**
Bivariate 1	Any failure in the past five weeks in guest room return temperature (variable no. 1)	1.1 (0.6, 2)
Bivariate 2	Any failure in the past five weeks in guest room supply temperature (variable no. 2)	1.6 (0.7, 3.6)
Bivariate 3	Any failure in the past five weeks at the primary test point for total disinfectant in cold water (variable no. 3)	1.5 (0.9, 2.4)
Bivariate 4	Any failure in the past five weeks at the primary test point for free disinfectant in cold water (variable no. 4)	1.1 (0.7, 1.7)
Bivariate 5	Any failure in the past five weeks at the primary test point for total disinfectant in hot water (variable no. 5)	3.8 (2, 7.2) *
Bivariate 6	Any failure in the past five weeks at the primary test point for free disinfectant in hot water (variable no. 6)	1.4 (0.9, 2.3)
Model 1	Sample collected from the hot water system	2.3 (1.5, 3.7) *
Model 1	Sample collected in the high season	0.8 (0.3, 1.9)
Model 1	Any failure in the past five weeks in guest room return temperature (variable no. 1)	1.5 (0.8, 3.1)
Model 1	Any failure in the past five weeks in guest room supply temperature (variable no. 2)	1.5 (0.5, 4.9)
Model 1	Any failure in the past five weeks at the primary test point for total disinfectant in cold water (variable no. 3)	1 (0.5, 2)
Model 1	Any failure in the past five weeks at the primary test point for cold water free disinfectant (variable no. 4)	0.8 (0.4, 1.5)
Model 1	Any failure in the past five weeks at the primary test point for total disinfectant in hot water (variable no. 5)	4.9 (2.2, 10.6) *
Model 1	Any failure in the past five weeks at the primary test point for hot water free disinfectant failure (variable no. 6)	0.8 (0.4, 1.6)
Model 1	Sample collected in 2020	2.4 (1.4, 4.3) *
Model 1	Sample collected in 2019	1.3 (0.7, 2.3)
Model 2	Sample collected from the hot water system	2.3 (1.5, 3.7) *
Model 2	Sample collected in the high season	0.8 (0.3, 1.9)
Model 2	Any failure in the past five weeks in guest room return temperature failure (variable no. 1)	1.6 (0.8, 3.2)
Model 2	Any failure in the past five weeks at the primary test point for total disinfectant in cold water (variable no. 3)	1 (0.5, 2)
Model 2	Any failure in the past five weeks at the primary test point for cold water free disinfectant (variable no. 4)	0.8 (0.4, 1.5)
Model 2	Any failure in the past five weeks at the primary test point in total disinfectant in hot water (variable no. 5)	4.7 (2.2, 10.2) *
Model 2	Any failure in the past five weeks at the primary test point for hot water free disinfectant (variable no. 6)	0.8 (0.4, 1.6)
Model 2	Sample collected in 2020	2.4 (1.4, 4.3) *
Model 2	Sample collected in 2019	1.2 (0.7, 2.3)
Model 3	Sample collected from the hot water system	2.3 (1.5, 3.7) *
Model 3	Sample collected in the high season	0.8 (0.3, 1.8)
Model 3	Any failure in the past five weeks in return temperature in guest room (variable no. 1)	1.6 (0.8, 3.2)
Model 3	Any failure in the past five weeks at the primary test point for total disinfectant in cold water (variable no. 3)	0.9 (0.5, 1.7)
Model 3	Any failure in the past five weeks at the primary test point for total disinfectant in hot water (variable no. 5)	4.6 (2.1, 10) *
Model 3	Any failure in the past five weeks at the primary test point for hot water free disinfectant (variable no. 6)	0.7 (0.4, 1.4)
Model 3	Sample collected in 2020	2.4 (1.3, 4.2) *
Model 3	Sample collected in 2019	1.2 (0.7, 2.3)
Model 4	Sample collected from the hot water system	2.4 (1.5, 3.7) *
Model 4	Sample collected in the high season	0.8 (0.3, 1.8)
Model 4	Any failure in the past five weeks in guest room return temperature (variable no. 1)	1.6 (0.8, 3.2)
Model 4	Any failure in the past five weeks at the primary test point for total disinfectant in hot water (variable no. 5)	4.5 (2.1, 9.5) *
Model 4	Any failure in the past five weeks at the primary test point for hot water free disinfectant failure (variable no. 6)	0.8 (0.4, 1.4)
Model 4	Sample collected in 2020	2.4 (1.3, 4.2) *
Model 4	Sample collected in 2019	1.3 (0.7, 2.3)
Model 5 [Final model]	Sample collected from the hot water system	2.3 (1.5, 3.7) *
Model 5 [Final model]	Sample collected in the high season	0.8 (0.3, 1.9)
Model 5 [Final model]	Any failure in the past five weeks in guest room return temperature (variable no. 1)	1.6 (0.8, 3.2)
Model 5 [Final model]	Any failure in the past five weeks at the primary test point for total disinfectant in cold water (variable no. 3)	0.9 (0.5, 1.6)
Model 5 [Final model]	Any failure in the past five weeks at the primary test point for total disinfectant in hot water (variable no. 5)	4.1 (2, 8.5) *
Model 5 [Final model]	Sample collected in 2020	2.4 (1.4, 4.3) *
Model 5 [Final model]	Sample collected in 2019	1.2 (0.7, 2.3)
Model 6	Sample collected from the hot water system	2.6 (1.7, 4) *
Model 6	Sample collected in the high season	0.8 (0.4, 1.8)
Model 6	Any failure in the past five weeks at the primary test point for total disinfectant in hot water (variable no. 5)	4.5 (2.2, 9.5) *
Model 6	Any failure in the past five weeks at the primary test point for hot water free disinfectant (variable no. 6)	0.9 (0.5, 1.6)
Model 6	Sample collected in 2020	2.3 (1.3, 4) *
Model 6	Sample collected in 2019	1.3 (0.7, 2.2)
Model 7	Sample collected from the hot water system	2.4 (1.5, 3.7) *
Model 7	Sample collected in the high season	0.8 (0.3, 1.8)
Model 7	Any failure in the past five weeks in guest room return temperature (variable no. 1)	1.6 (0.8, 3.2)
Model 7	Any failure in the past five weeks at the primary test point for total disinfectant in hot water (variable no. 5)	3.9 (1.9, 7.9) *
Model 7	Sample collected in 2020	2.4 (1.4, 4.2) *
Model 7	Sample collected in 2019	1.3 (0.7, 2.3)
* significant.		

## Appendix D. Overlap in Failures

**Guest Room Return Water Temperature (Variable No. 1)**	**Primary Test Point for Total Disinfectant in Cold Water** **(Variable No. 3)**	**Primary Test Point for Total Disinfectant in Hot Water** **(Variable No. 5)**	**Number**
No failure or NA	No failure or NA	No failure or NA	2316
No failure or NA	No failure or NA	Failure	1226
No failure or NA	Failure	No failure or NA	67
No failure or NA	Failure	Failure	1010
Failure	No failure or NA	No failure or NA	350
Failure	No failure or NA	Failure	204
Failure	Failure	No failure or NA	24
Failure	Failure	Failure	238

## Appendix E. Primary Analysis Repeated for Any *Legionella* Detection (SG1 or Non-SG1) as the Outcome

### Appendix E.1. Odds Ratios Comparing Legionella Detection to Failure to Detect *

**Variable**	**OR (95% CI)**
Any failure in the past five weeks regarding return hot water temperature in guest rooms (variable no. 1)	1.3 (0.8, 2)
Any failure in the past five weeks at the primary test point for total disinfectant levels in cold water (variable no. 3)	1 (0.7, 1.4)
Any failure in the past five weeks at the primary test point for total disinfectant levels in hot water (variable no. 5)	2.5 (1.7, 3.7)
Sample collected from the hot water system	2.3 (1.8, 3)
Sample collected in 2020	2.6 (1.9, 3.7)
Sample collected in 2019	1.1 (0.8, 1.6)
Sample collected in2018	
Sample collected in the high season	0.9 (0.5, 1.6)
* significant, at alpha = 0.05.	

### Appendix E.2. Performance Metrics

**Variable (Any Failure in the Past Five Weeks)**	**PPV**	**NPV**	**Sensitivity**	**Specificity**
Guest room supply temperature failure (variable no. 1)	14.0%	85.1%	5.5%	94.1%
Return temperature in guest room failure (variable no. 2)	16.8%	85.6%	22.6%	80.4%
Primary test point for total disinfectant failure in cold water (variable no. 3)	20.4%	88.4%	45.6%	69.9%
Primary test point for cold water free disinfectant failure (variable no. 4)	17.6%	88.0%	52.9%	58.3%
Primary test point for total disinfectant failure in hot water (variable no. 5)	18.0%	92.1%	80.9%	37.6%
Primary test point for hot water free disinfectant failure (variable no. 6)	17.6%	88.7%	61.2%	51.4%
